# Punctate inner choroidopathy immediately after COVID-19 infection: a case report

**DOI:** 10.1186/s12886-022-02514-8

**Published:** 2022-07-07

**Authors:** Manabu Miyata, Sotaro Ooto, Yuki Muraoka

**Affiliations:** Department of Ophthalmology and Visual Sciences, Kyoto University Graduate School of Medicine, Shogoin Kawahara-cho 54, Sakyo-ku, Kyoto, 606-8507 Japan

**Keywords:** COVID-19, Oral prednisolone, Punctate inner choroidopathy

## Abstract

**Background:**

Punctate inner choroidopathy (PIC) is a rare idiopathic inflammatory multifocal chorioretinopathy. Although the etiology of PIC is unknown, it is proposed to be an autoimmune disease that arises in the context of polygenic susceptibility triggered by an environmental stimulus, such as infection. We reported a case of PIC immediately after COVID-19 infection.

**Case presentation:**

A 25-year-old woman complained of blurred vision in the right eye six days after the symptoms of COVID-19 infection first appeared. The patient visited our hospital and underwent comprehensive ophthalmological examination 18 days after the initial COVID-19 symptoms. Based on the characteristic fundus features observed with multimodal imaging, retinal specialists made a diagnosis of PIC. The patient was affected with high myopia. As her general COVID-19 symptoms disappeared, the patient was prescribed oral prednisolone 30 mg/day for 14 days to treat PIC. Fundus abnormality decreased and her ocular symptoms improved. No side effects were observed, including the recurrence of general COVID-19 symptoms.

**Conclusion:**

We experienced an extremely rare case of PIC immediately after COVID-19 infection and showed the potential safety and effectiveness of oral prednisolone in treating PIC in the active phase after the disappearance of the general COVID-19 infection symptoms.

## Background

Punctate inner choroidopathy (PIC), first described by Watzki in 1984 [1], is a rare idiopathic inflammatory multifocal chorioretinopathy that most commonly affects young myopic women [2]. Although the etiology of PIC is unknown, it is proposed to be an autoimmune disease that arises in the context of polygenic susceptibility triggered by an environmental stimulus, such as infection, immunization, or stress [2]. A recent case report showed a young Caucasian woman with bilateral recurrent PIC 3 weeks after COVID-19 infection [3]. We describe the case of a young Asian woman with new-onset PIC 6 days after the symptoms of COVID-19 infection.

## Case presentation

A 25-year-old woman complained of blurred vision in the right eye six days after the symptoms of COVID-19 infection, i.e., fever, first appeared. The infection was diagnosed by reverse transcriptase polymerase chain reaction. Previous medical history included a neurosurgical procedure to treat Moyamoya disease by anastomosis of the left superficial temporal artery and left middle cerebral artery performed at 18 years of age.

**Fig. 1:**
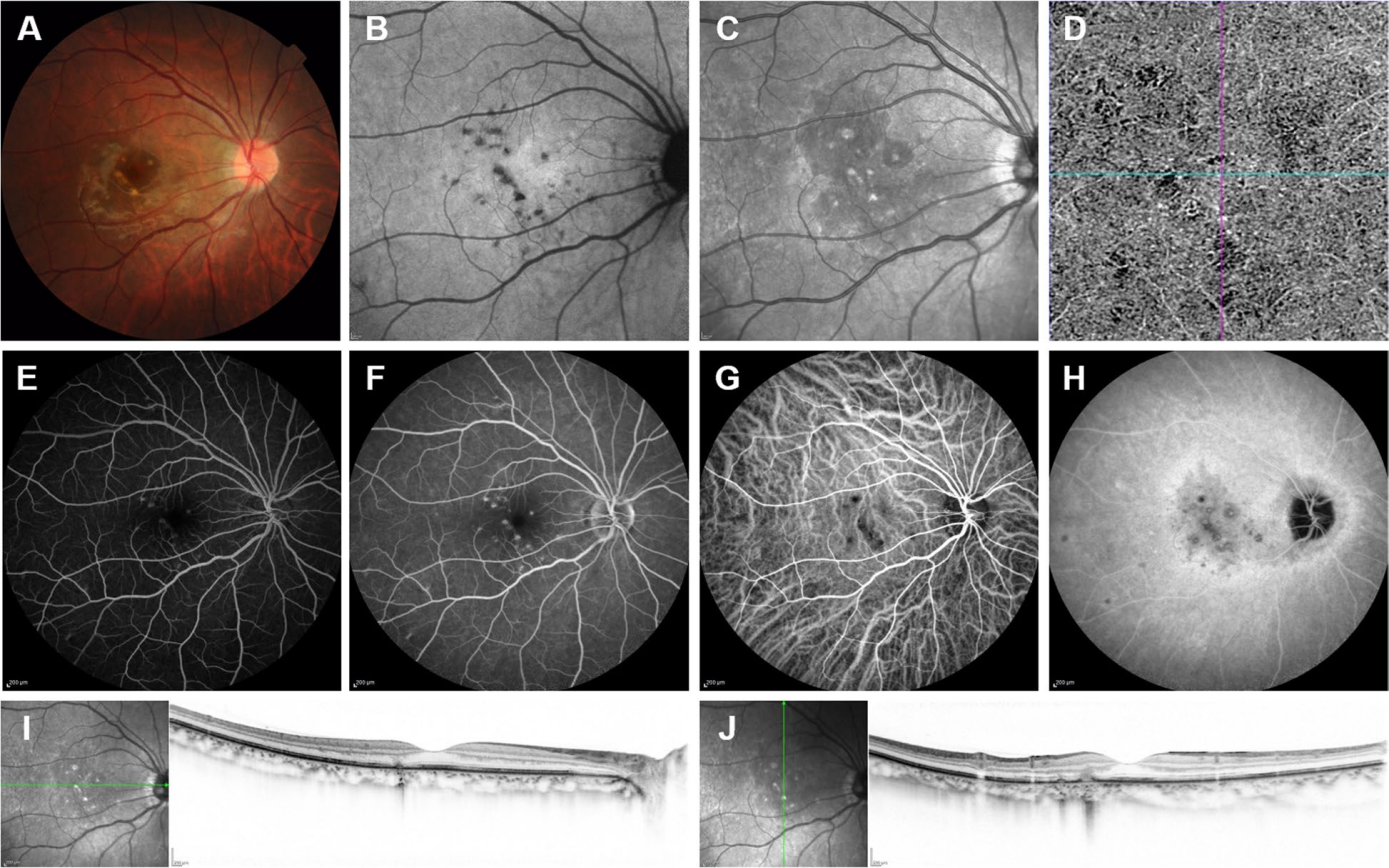
Multimodal fundus images in active phase. **A** A color fundus photograph shows multifocal, small, yellow-white spots in the extrafovea. **B** A fundus autofluorescence image shows punctate hypo-autofluorescent lesions. **C** A fundus infrared image shows punctate hyper-reflective lesions and a relatively broad area of hypo-reflective lesions around them. **D** An optical coherence tomography (OCT) angiography image of a 3 × 3 mm^2^ section of the outer retina to the choriocapillaris layer shows several deficits of the choriocapillaris compatible with focal ischemic areas and no relevant choroidal neovascularization. **E**, **F** Fluorescent fundus angiography images obtained at early (17 s) and late phases (15 min 13 s) show impregnation of the dye with staining phenomena and their increase in intensity without leakage phenomena, respectively. **G**, **H** Indocyanine green fundus angiography images obtained at early (17 s) and late phases (15 min 13 s) show macular hypo-cyanescent lesions and clarity of their margins, respectively. **I**, **J** Horizontal- and vertical-scan OCT images through the fovea show focal elevation of the retinal pigment epithelium (RPE) and breakthrough of the RPE with the accumulation of parafoveal subretinal hyperreflective material, referable to inflammatory elements, respectively. Infrared images, indicating scan line, showed multiple hyperreflective spots

The patient visited our hospital and underwent comprehensive ophthalmological examination 18 days after the initial COVID-19 symptoms. Color fundus photography revealed multifocal, small, yellow-white spots in the extrafovea (Fig. 1A). Fundus autofluorescence showed punctate hypo-autofluorescent lesions (Fig. 1B), and a fundus infrared image showed punctate hyper-reflective lesions surrounded by a relatively broad area of hypo-reflective lesions (Fig. 1C). Optical coherence tomography (OCT) angiography of the outer retina to the choriocapillaris layer showed several deficits of the choriocapillaris compatible with focal ischemic areas and no relevant choroidal neovascularization (CNV, Fig. 1D). Punctate hyper-fluorescent lesions were observed on fluorescein fundus angiography (Fig. 1E and F). Indocyanine green fundus angiography showed choroidal hypo-cyanescent lesions and slightly hypo-cyanescent lesions (Fig. 1G and H). OCT revealed focal elevation of the retinal pigment epithelium (RPE) and breakthrough of the RPE with the accumulation of parafoveal subretinal hyperreflective material, referable to inflammatory elements, respectively (Fig. 1I and J). Furthermore, there was no vitreous cell on OCT images. Slit-lamp biomicroscopy revealed no cell in the anterior chamber. Based on these characteristic fundus features observed with multimodal imaging, retinal specialists made a diagnosis of PIC. The case was classified as stage III, based on the staging described by Zhang, et al. [4]. The patient was affected with high myopia (axial length, 27.71 mm; refractive error in spherical equivalence, − 9.25 diopters in the right eye).

**Fig. 2:**
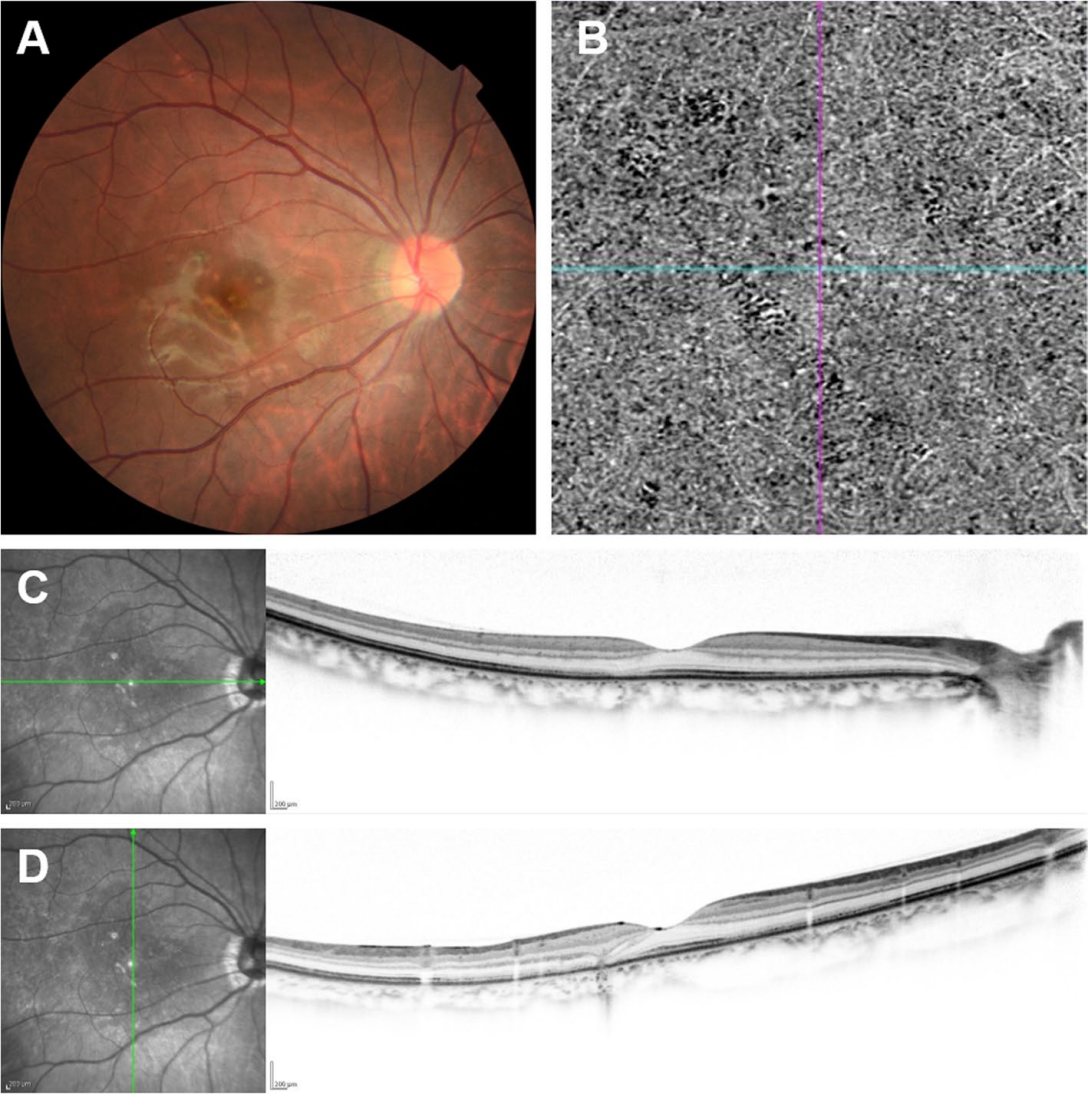
Multimodal fundus images 2 weeks after initiating oral prednisolone. **A** A color fundus photograph shows fading of the multiple yellow-white spots. **B** Optical coherence tomography (OCT) angiography of a 3 × 3 mm^2^ section at the outer retina to the choriocapillaris layer shows reduction in the area of deficits of the choriocapillaris. Choroidal neovascularization was not observed. **C** A horizontal-scan OCT image showed that a focal elevation of the retinal pigment epithelium (RPE) disappeared. However, outer retinal damage was persistent at the lesion. An infrared image, indicating scan line, shows reduction in the number of hyperreflective spots. **D** A vertical-scan OCT image reveals the disappearance of the smaller focal elevation of the RPE. Although the bigger focal elevation remained, it became smaller. Outer retinal damage was persistent at these lesions

As her general COVID-19 symptoms disappeared, the patient was prescribed oral prednisolone 30 mg/day for 14 days to treat PIC. Fundus abnormality decreased and her ocular symptoms improved (Fig. 2). We subsequently reduced oral prednisolone to 15 mg/day for the next 14 days, to 10 mg/day for the next 14 days, and to 5 mg/day for the next 28 days. No side effects were observed, including the recurrence of general COVID-19 symptoms.

## Discussion and conclusions

To the best of our knowledge, this is the first report of a young female patient affected with high myopia and unilateral new-onset PIC associated with COVID-19 infection. A previous report showed unilateral multifocal choroiditis in a COVID-19 positive patient [5]. Multifocal choroiditis and PIC have been included under the umbrella term of “white spot syndromes.” Eyes with PIC have scarring mostly in the posterior pole and little to no vitreous inflammation. They generally have a good visual prognosis unless CNV develops. However, eyes with multifocal choroiditis have vitreous inflammation, scarring mostly in the mid-peripheral retina [6]. Therefore, we diagnosed our case as PIC.

A recent case report showed a 29-year Caucasian woman with bilateral PIC recurrence three weeks after COVID-19 infection [3]. She was affected with bilateral PIC complicated by secondary CNV in the left eye two years before the recurrence. The disease was in a clinically quiescent phase for two years. However, COVID-19 infection could have been a trigger for PIC recurrence. The etiology of white spot syndromes, including PIC, was proposed to be autoimmune disease [2]. Therefore, our case has the necessity for a further follow-up.

Further, few case reports have shown other types of choroiditis and retinitis following not only COVID-19 infection but also vaccination. Acute macular neuroretinopathy occurred in a 71-year-old woman 14 days after COVID-19 infection [7] and in a 27-year-old woman two days after administration of the COVID-19 vaccine [8]. Furthermore, multiple evanescent white dot syndrome occurred in a 17-year-old man two months and ten days after COVID-19 infection [9] and in a 30-year-old woman three days after the second dose of the COVID-19 vaccine [10]. Serpiginous choroiditis occurred in a 41-year-old woman after COVID-19 infection [11]. Aforementioned multifocal choroiditis was reported in a 34-year-old man one week after the second dose of the COVID-19 vaccine [12]. Hence, both COVID-19 infection and vaccination may be rarely associated with autoimmune choroiditis or retinitis.

There is no literature to show treatment of PIC in the active phase, to our knowledge. Hence, we conventionally initiate 2-week oral prednisolone treatment of 30 mg/day and reduce it in accordance with disease status. This treatment was effective in this case, similar to the findings of a previous case report of PIC with CNV [13]. The report commented that oral steroid administration in PIC may help improve vision more rapidly. Our case revealed subjective visual improvement and no CNV occurrence after oral prednisolone without general side effects.

This report has some limitations. First, the temporal relationship between PIC and COVID-19 infection could be by chance. However, PIC is proposed to be an autoimmune disease caused by an infection. Since COVID-19 infection is reported to correlate with the autoimmunity markers [14], we considered that COVID-19 infection may have affected her immunity and induced PIC as the blurred vision occurred because six days after the general COVID-19 symptoms appeared. Second, there was no comparison between the administration of oral prednisolone and the natural course. PIC might spontaneously resolve. However, no side effects of prednisolone appeared in this case; this would be a significant finding.

In conclusion, we experienced an extremely rare case of PIC and showed the potential safety and effectiveness of oral prednisolone in treating PIC in the active phase after the disappearance of COVID-19 infection symptoms.

## Data Availability

All relevant findings in the patient describe here are included in this report.

## Abbreviations

CNV: Choroidal neovascularization
OCT: Optical coherence tomography
PIC: Punctate inner choroidopathy
RPE: Retinal pigment epithelium
